# Unusual Repertoire of Vocalizations in the BTBR T+tf/J Mouse Model of Autism

**DOI:** 10.1371/journal.pone.0003067

**Published:** 2008-08-27

**Authors:** Maria Luisa Scattoni, Shruti U. Gandhy, Laura Ricceri, Jacqueline N. Crawley

**Affiliations:** 1 Laboratory of Behavioral Neuroscience, National Institute of Mental Health, Bethesda, Maryland, United States of America; 2 Behavioural Neurosciences Section, Department of Cell Biology and Neurosciences, Istituto Superiore di Sanita', Rome, Italy; Centre National de la Recherche Scientifique, France

## Abstract

BTBR T+ tf/J (BTBR) is an inbred mouse strain that displays social abnormalities and repetitive behaviors analogous to the first and third diagnostic symptoms of autism. Here we investigate ultrasonic vocalizations in BTBR, to address the second diagnostic symptom of autism, communication deficits. As compared to the commonly used C57BL/6J (B6) strain, BTBR pups called more loudly and more frequently when separated from their mothers and siblings. Detailed analysis of ten categories of calls revealed an unusual pattern in BTBR as compared to B6. BTBR emitted high levels of harmonics, two-syllable, and composite calls, but minimal numbers of chevron-shaped syllables, upward, downward, and short calls. Because body weights were higher in BTBR than B6 pups, one possible explanation was that larger thoracic size was responsible for the louder calls and different distribution of syllable categories. To test this possibility, we recorded separation calls from FVB/NJ, a strain with body weights similar to BTBR, and 129X1/SvJ, a strain with body weights similar to B6. BTBR remained the outlier on number of calls, displaying low numbers of complex, upward, chevron, short, and frequency steps calls, along with high harmonics and composites. Further, developmental milestones and growth rates were accelerated in BTBR, indicating an unusual neurodevelopmental trajectory. Overall, our findings demonstrate strain-specific patterns of ultrasonic calls that may represent different lexicons, or innate variations in complex vocal repertoires, in genetically distinct strains of mice. Particularly intriguing is the unusual pattern of vocalizations and the more frequent, loud harmonics evident in the BTBR mouse model of autism that may resemble the atypical vocalizations seen in some autistic infants.

## Introduction

Vocal communication in animals has been extensively documented for many species, including songbirds, whales, and dolphins [1]–[3]. Adult rodents emit vocalizations during aggressive, play, mating interactions, and in response to some stressors [4]–[8]. Infant mice and rats emit ultrasonic vocalizations (USVs) which serve to elicit pup retrieval by the parents and maternal licking and crouching behaviors [9]–[16], suggesting that these calls play an important role in social communication between mother and infant. While rat calls in the 22 kHz and 50 kHz range have been extensively described [5], [17]–[20], specific elements of mouse vocal emissions have only recently begun to be characterized [8], [21]–[24].

Abnormal reciprocal social interactions and communication deficits are two of the three diagnostic symptoms of autism [25]. We hypothesize that USVs may be a measure of social communication in mice [15], [26]–[28]. Reduced or unusual USVs in mice may offer a useful assay with reasonable face validity to the second diagnostic symptom of autism, impaired communication. A standard test for vocalizations in mice, the ultrasonic distress call of pups separated from the mother or removed from the nest [29]–[31], has been used to investigate the number of calls emitted by separated pups in mouse models of autism spectrum disorders [15], [32]–[39].

We previously reported on an inbred strain of mice, BTBR T+tf/J (BTBR), that displays several traits relevant to autism, including reduced social approach in adults, reduced reciprocal social interactions in juveniles and adults, decreased social transmission of food preference, and high repetitive self grooming when compared to C57BL/6J (B6), a standard inbred strain commonly used in behavioral genetics [40]–[44]. To address the hypothesis that BTBR also display communication deficits, and that autism-like phenotypes in mice can be detected at early developmental stages, we analyzed neonatal USV patterns (Cohort 1) and behavioral and somatic development (Cohort 2) in BTBR mice throughout the first two weeks of postnatal life. The primary goal of the present studies was to detect any unusual components of vocalizations in BTBR at infant stages, relevant to the absence of crying, and the unusual guttural grunts and squeals, reported for some babies that were later diagnosed with autism [45], [46].

To define “unusual” BTBR vocalization patterns in the absence of normative sonographs of USVs across inbred strains of mice, we compared USVs in BTBR versus three control strains typically used in behavioral genetics and as backgrounds for breeding genetically modified mice. B6 represented a widely-used standard control with high levels of social behaviors [47] and low USV calling rate [10], [48]–[52]. FVB/NJ was selected as a strain with high body weights similar to BTBR, to control for the possibility that larger lung capacity could be responsible for unusual vocalizations. In addition, FVB/NJ displays high levels of sociability in the social approach apparatus, comparable to B6. 129X1/SvJ (129X1) was selected as genetically close to BTBR on the mouse phylogenetic tree [53] but with body weights similar to B6.

Our findings demonstrate a comparable use of ten different categories of calls that may represent a similar “language” in three inbred mouse strains. In contrast, the BTBR strain showed an unusual pattern of vocalizations, emitted only four out of ten categories of calls, and displayed more frequent, loud harmonics.

## Results

### Vocalizations

#### Pup separation vocalizations

**Figure 1:**
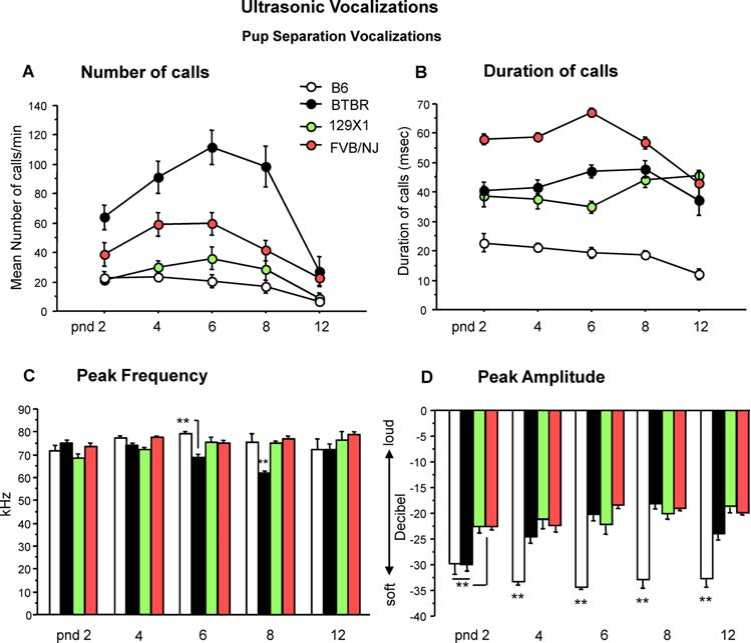
Ultrasonic vocalizations (USVs) in BTBR, B6, 129X1 and FVB/NJ pups. A) Number and B) Duration of vocalizations on postnatal day (pnd) 2, 4, 6, 8 and 12 in response to social separation during a five minute session. Significant strain differences were detected across five days of testing. C) Peak frequency and D) amplitude of USVs analyzed on each day of testing. Data are expressed as mean±SEM of calls. Figures 1–6 present results from Cohort 1, N = 20 mice per strain, representing one male and one female from each of 10 litters per strain. *p<.05 and **p<.01 for the comparisons between strains.

Measurements of ultrasonic vocalizations in separated pups of the four strains [postnatal day (pnd) 2 to 12] detected prominent differences between the BTBR pups versus the other three strains. Total number of vocalizations varied across strains [F (3,36) = 38.89, p<.0001] (Figure 1A). On pnd 2, 4, 6 and 8, BTBR pups emitted significantly more calls [strain×age interaction: F(12,144) = 3.54, p<.0001; Newman-Keuls p<.0001 for BTBR versus B6 and 129X1 at pnd 2, 4, 6 and 8; Newman-Keuls p<.005 for BTBR vs FVB/NJ at pnd 4, 6 and 8]. Duration of vocalizations differed across strains [F(3,36) = 129.17, p<.0001](Figure 1B). BTBR pups emitted longer calls than the B6 mice throughout the first two postnatal weeks [strain×age interaction: F(12,144) = 5.02, p<.0001; Newman-Keuls p<.0001 for BTBR versus B6 at any age considered]. BTBR showed call durations similar to 129X1 except on pnd 6 [Newman-Keuls p = .02]. In contrast, BTBR had significantly lower call duration than FVB/NJ pups from pnd 2 to 8 [Newman-Keuls p<.001 at pnd 2, 4, 6; p<.01 at pnd 8]. Average peak frequency varied across strains [F(3,36) = 9.14, p<.0001] (Figure 1C). On pnd 6 and 8, BTBR pups emitted calls with lower frequency [strain×age interaction: F(12,144) = 3.63, p<.0001; Newman-Keuls at pnd 6: p = .05 for BTBR versus B6; Newman-Keuls at pnd 8: p = .005 for BTBR vs all strains]. Average peak amplitude also showed a significant difference across strain [F(3,36) = 86.94, p<.0001]. On pnd 2, BTBR and B6 pups emitted softer calls than FVB/NJ and 129X1 [strain×age interaction: F(12,144) = 4.68, p<.0001; Newman-Keuls p<.001 for BTBR and B6 versus FVB/NJ and 129X1]. The B6 pups continued to emit soft calls while the BTBR pups began to call more loudly, starting on pnd 4 through pnd 12, than the age-matched B6 [Newman-Keuls p<.0001] (Figure 1D).

No differences were detected in body temperatures of B6, BTBR and FVB/NJ as measured after each separation test. Only 129X1 pups showed a lower temperature at pnd 12 in comparison to the other strains [strain×postnatal days interaction: F (12,144) = 4.414, p<.0.001; Newman-Keuls p<.001] (see Table S1 in the online Supporting Information).

#### Classification of ultrasonic vocalizations into distinct categories

**Figure 2 pone-0003067-g002:**
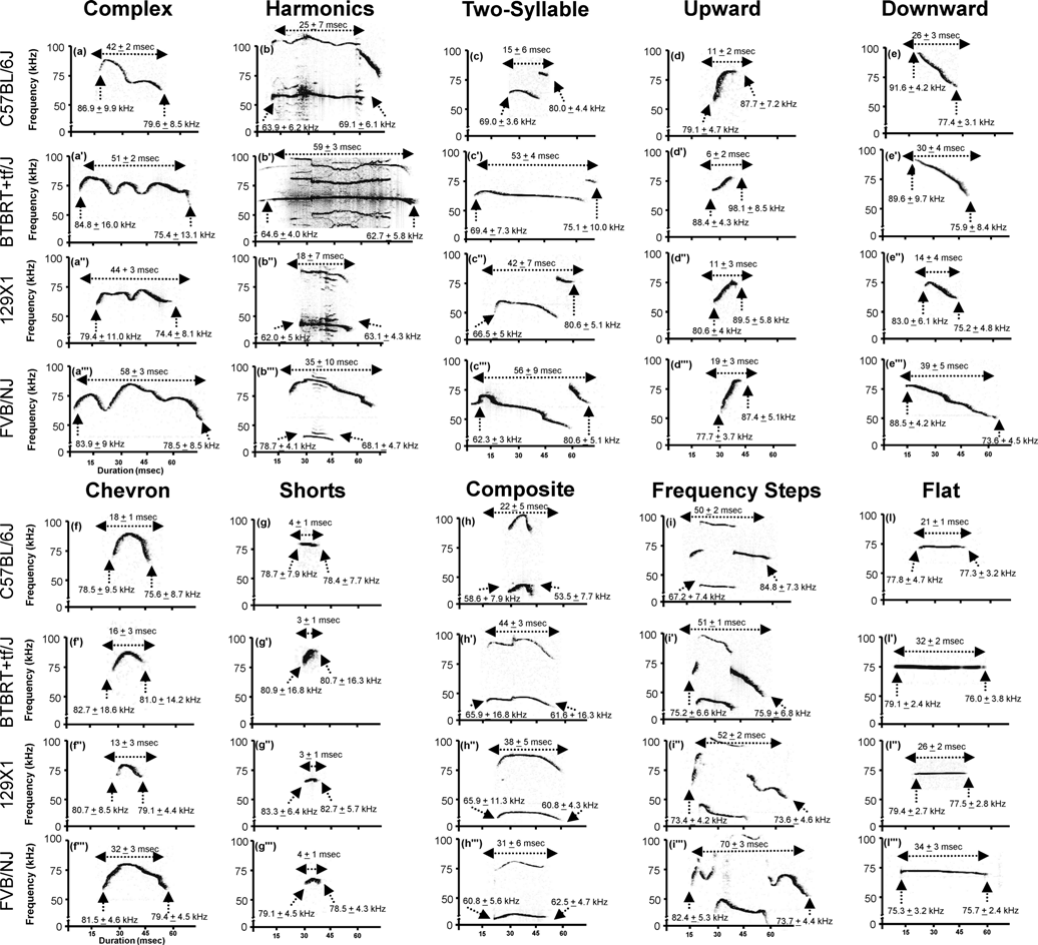
Typical sonograms of ultrasonic vocalizations, classified into ten distinct categories of calls emitted by (a–l) B6, (a'–l') BTBR, (a''–l'') 129X1, and (a'''–l''') FVB/NJ mice. Descriptive statistics (mean±SEM) are given for the duration of each call type, as well as the beginning and ending dominant frequency.

Examples of sonograms typical for each pattern category are shown in Figures 2 and S1. Audiofiles illustrating each call are available in the online Supporting Information (Sounds S1, S2, S3, S4, S5, S6, S7, S8, S9 and S10), as described at the end of the Methods section below.

**Figure 3 pone-0003067-g003:**
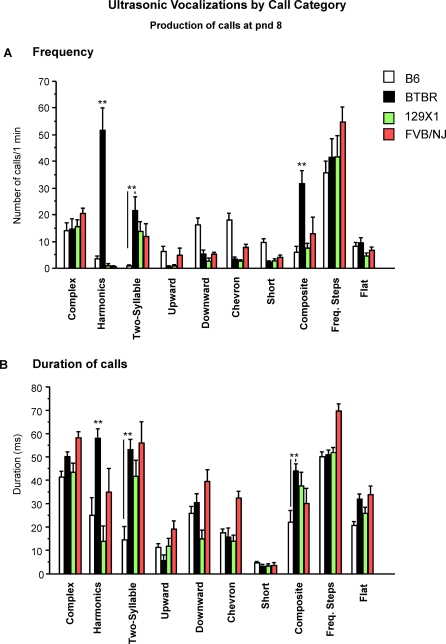
Production of ultrasonic vocalizations by call category at postnatal day 8. A) Frequency and B) Duration of ultrasonic vocalizations, during a total of twenty sonograms per strain, each of one minute duration. Harmonic and composite calls frequency and harmonics duration: **p<.01 BTBR compared to B6, 129X1 and FVB/NJ pups; Two-syllables frequency and duration and composite call duration: **p<.01 BTBR compared to B6 pups.

Figure 3 illustrates strain-dependent effects on both frequency [F(3,76) = 29.62, p<.0001] and duration of calls [F(3,76) = 19.33, p<.0001]. BTBR emitted a significantly higher number of harmonic-modulated calls [strain×calls subtype interaction: F(27,684) = 8.87, p<.0001; Newman-Keuls p<.001 for BTBR vs all strains], two-syllable calls [Newman-Keuls p<.05 for BTBR vs B6], and composite calls [Newman-Keuls p<.001 for BTBR vs all strains].

BTBR pups also emitted longer harmonic calls than the other strains [strain×calls subtype interaction: F(27,684) = 4.67, p<.0001; Newman-Keuls p<.001]. The two-syllable and composite calls emitted by BTBR pups were of longer duration in comparison to the same subtypes emitted by age-matched B6 [p<.01].

#### Pattern of sonographic structure among strains

**Figure 4 pone-0003067-g004:**
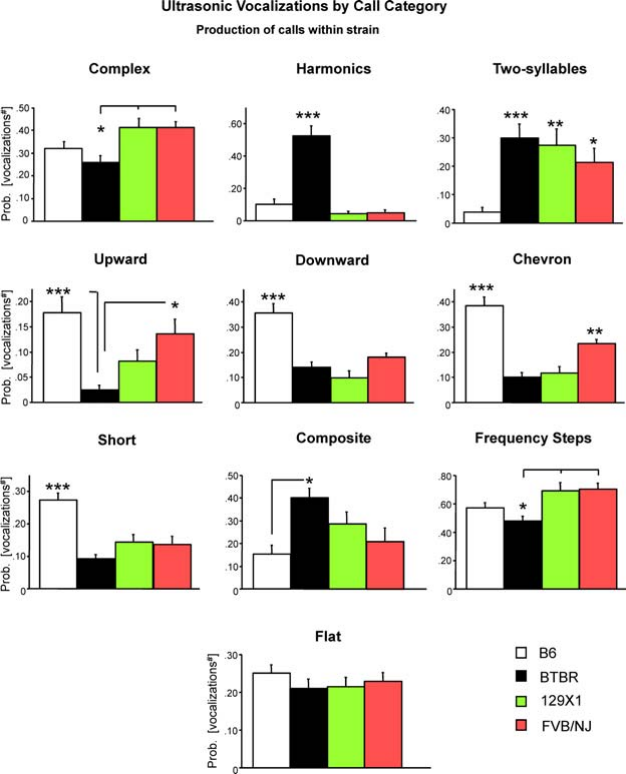
Production of calls within strain. Probability of producing calls from each of the ten categories of USV. ^#^Data were expressed by angular transformation. Number of calls analyzed: B6 = 2333; BTBR = 3633; 129X1 = 1806; FVB/NJ = 2575 collected from 20 sonograms per strain representative of each pup tested at pnd 8. *p<.05; **p<.01 and ***p<.001 for the comparisons between strains.

**Figure 5 pone-0003067-g005:**
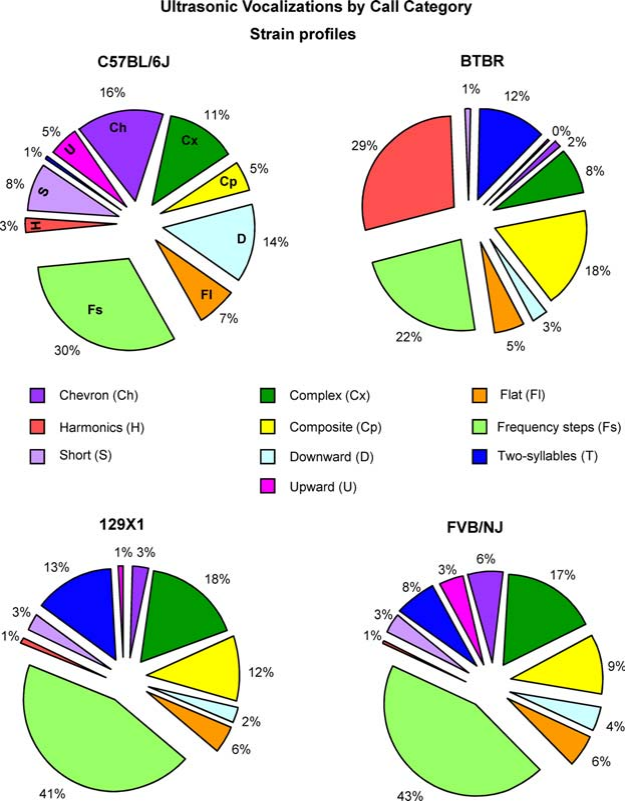
Strain profiles. Pie graphs show the percentages of the different call categories within strain. Percentages were calculated in each strain as number of calls in each category for each subject/total number of calls analyzed in each subject. Number of total calls analyzed: B6 = 2333; BTBR = 3633; 129X1 = 1806; FVB/NJ = 2575.

Proportions of calls within each category are shown in Figures 4 and 5.

The call distributions differed across strains [F(3,76) = 4.60, p<.005] and category [strain×category interaction: F(27,684) = 11.89, p<.0001]. As shown in Figure 5, B6, 129X1 and FVB/NJ pups emitted a wide spectrum of call categories while BTBR pups emitted a narrower spectrum of call categories. BTBR displayed high prevalence in production of harmonic, frequency steps, composite and two-syllable calls, along with low prevalence in production of upward, chevron, short, and downward calls, indicating an apparently unusual pattern of vocalizations as compared to the three other strains tested in the present study.

When analyzing each USV category separately, a strain-dependent effect was found on the probability of producing calls from nine of the ten call categories (number of calls in each category for each subject/total number of calls analyzed in each subject) [complex: F(3,76) = 5.38, p<.005; harmonics: F(3,76) = 39.76, p<.0001; two-syllable: F(3,76) = 6.81, p<.0005; upward: F(3,76) = 7.17, p<.0005; downward: F(3,76) = 17.10, p<.0001; chevron: F(3,76) = 27.91, p<.0001; short: F(3,76) = 12.66, p<.0001; composite: F(3,76) = 4.82, p<.005; frequency steps: F(3,76) = 6.29, p = .0007] (Figure 4). The probability of emitting flat calls did not differ across strains [F(3,76) = 0.62, p = .59].

BTBR pups emitted more harmonics than B6, 129X1 and FVB/NJ pups [Newman-Keuls with Bonferroni correction p<.001] and more composite [p<.05] and two-syllable calls than B6 pups [p<.001] (Figure 4). BTBR pups emitted less complex and frequency steps than 129X1 and FVB/NJ pups [p<.05] and less upward calls than B6 [p<.001] and FVB/NJ pups [p<.05]. B6 pups emitted more downward, chevron and short calls than the other strains [Newman-Keuls with Bonferroni correction p<.001]. Moreover, B6 pups emitted less two-syllable calls than the other strains [p<.001 vs BTBR; p<.01 vs 129X1 and p<.05 vs FVB/NJ].

### Developmental Milestones

#### Body weight

**Figure 6:**
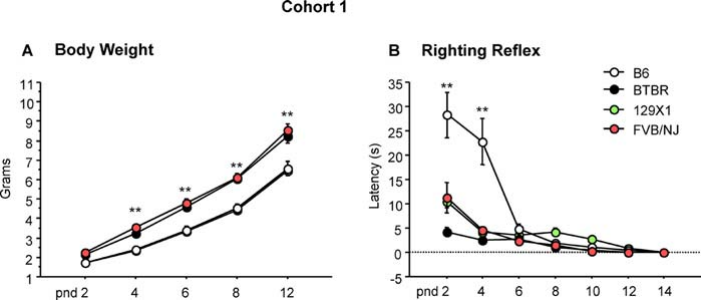
A) Body weights and B) Righting reflex latencies in animals that were tested for ultrasonic vocalizations (cohort 1). Body weights were higher in BTBR and FVB/NJ compared to B6 and 129X1 through pnd 4 to 12. **p<.01. Pups acquired the righting reflex response at different rates, with BTBR, 129X1 and FVB/NJ showing shorter latencies than B6 starting at pnd 2. **p<.01.

Body weight, measured from postnatal days (pnd) 2 to 12, immediately before the start of the ultrasonic vocalization test, differed significantly between BTBR, B6, 129X1 and FVB/NJ pups in the first cohort of mice used for ultrasonic vocalizations [strain effect: F(3,36) = 18.22, p<.0001]. BTBR and FVB/NJ pups had significantly greater weight gains starting from pnd 4 through pnd 12 [strain×age interaction: F(12,144) = 4.35, p<.0001; Newman-Keuls p<.0001 for BTBR and FVB/NJ versus B6 and 129X1 pups, Figure 6A].

**Figure 7:**
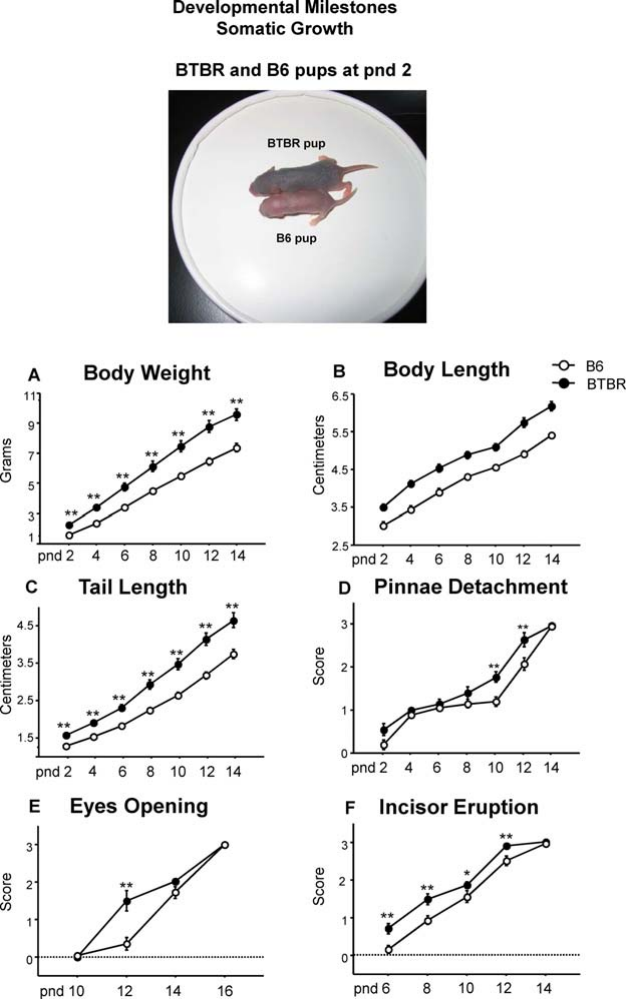
Somatic growth. Representative photograph of the body size differences between a pnd 2 BTBR (upper pup) and a pnd 2 B6 (lower pup). Analysis of the markers of somatic growth revealed that BTBR displayed accelerated development as compared to B6 on A) body weight, B) body and C) tail length, D) pinnae detachment, E) opening of the eyes and F) incisor eruption. Figures 7–10 present results from Cohort 2, N = 20 B6 and N = 20 BTBR, representing one male and one female from each of 10 litters per strain. ** p<.01 and *p<.05, for B6 vs. BTBR.

Body weights were also measured in a second cohort of mice used for the full set of developmental milestones. ANOVA revealed a significant difference in body weight of BTBR in comparison to B6 pups [strain effect: F(1,18) = 22.9, p<.0001]. Replicating the body weight difference seen in Cohort 1, BTBR mice were significantly heavier than B6 on pnd 4, 6, 8, and 12 [strain×age interaction: F(6,108) = 13.4, p<.0001; Newman-Keuls p<.001 for BTBR versus B6] (Figure 7A).

#### Righting reflex

**Figure 8 pone-0003067-g008:**
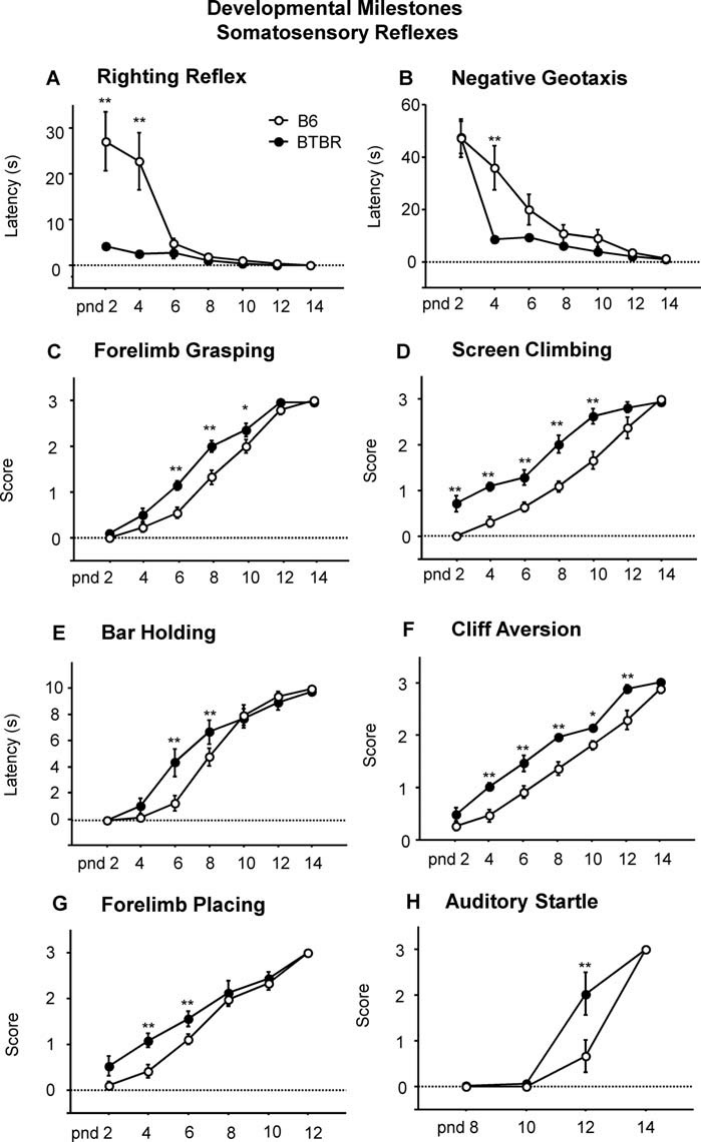
Somatosensory reflexes. Accelerated development was seen in BTBR as compared to B6 on A) righting reflex, B) negative geotaxis, C) forelimb grasping, D) screen climbing, E) bar holding, F) cliff aversion, G) forelimb placing and H) auditory startle. **p<.01 and *p<.05 for B6 vs. BTBR.

The righting reflex, measured as latency to turn back onto all four paws when placed on the back, was tested at pnd 2 to 14 in all four strains in the first cohort used for ultrasonic vocalizations. Righting reflex latencies differed significantly between B6 and the other strains [strain×age interaction: F(18,216) = 6.88, p<.0001; Newman-Keuls p<.0001 for B6 vs all strains at pnd 2 and 4] (Figure 6B). Righting reflex latencies were similarly measured in the second cohort of BTBR and B6, used for the full set of developmental milestones. A significant difference was detected between B6 and BTBR pups at pnd 2 and 4 [strain×age interaction: F(6,108) = 9.8, p<.0001; Newman-Keuls p<.01](Figure 8A).

#### Additional developmental milestones measured in the second cohort of B6 and BTBR

Analysis of markers of somatic growth (Figure 7) revealed that BTBR pups had an accelerated development compared to B6 pups on a) body weight, F(1,18) = 22.9, p<.0001; b) body length, F(1,18) = 57.6, p<.0001; c) tail length, F(1,18) = 26.8, p<.0001; d) pinnae detachment, F(1,18) = 8.0, p = .01; e) eye opening, F(1,18) = 10.6, p<.005; f) incisor eruption, F(1,18) = 12.7, p<.005. Post hoc comparisons revealed a significant difference in tail length at every age considered [strain×age interaction: F(6,108) = 8.8, p<.0001; Newman-Keuls p<.001 for BTBR versus B6]. Both BTBR and B6 pups had complete pinnae detachment by pnd 14 but the BTBR pups showed a significantly faster rate of detachment starting from pnd 10 through 12 [strain×age interaction: F(6,108) = 3.4, p<.005; Newman-Keuls p<.001 for BTBR versus B6 at pnd 10 and 12]. Opening of the eyes also occurred earlier in the BTBR pups. At pnd 12, B6 animals still had their eyes closed, while BTBR pups had eyes partially open [strain×age interaction: F(3,54) = 10.9, p<.0001; Newman-Keuls p<.001 for BTBR versus B6]. The same trend of early development in BTBR pups was noticed for incisor eruption. Post hoc comparisons revealed that BTBR pups showed faster incisor growth between pnd 6 and 12 [strain×age interaction: F(4,72) = 2.5, p = .05; Newman-Keuls p<.01 for BTBR vs B6 at pnd 6, 8 and 12; Newman-Keuls p<.05 at pnd 10].

BTBR pups were able to right themselves as early as pnd 2 [strain×age interaction: F(6,108) = 9.8, p<.0001; Newman-Keuls p<.01 for BTBR vs B6 at pnd 2 and 4] (Figure 8A). B6 first showed the righting reflex at pnd 6. BTBR and B6 pups acquired the negative geotaxis response at different rates, with BTBR showing lower latencies starting at pnd 4 [strain×age interaction: F(6,108) = 5.1, p<.001; Newman-Keuls p<.01 for BTBR vs B6] (Figure 8B).

Accelerated development in BTBR as compared to B6 was seen in c) forelimb grasping: F(1,18) = 11.1, p<.005; d) screen climbing: F(1,18) = 22.0, p<.001; f) cliff aversion: F(1,18) = 22.2, p<.001); g) forelimb placing: F(1,18) = 6.0, p<.05. Differing rates of acquisition of these reflexes (strain×age interaction) were detected: c) forelimb grasping: F(6,108) = 3.8, p<.005; Newman-Keuls p<.01 for BTBR versus B6 at pnd 6 and 8; Newman-Keuls p<.05 for BTBR versus B6 at pnd 10; d) screen climbing: F(6,108) = 4.7, p<.001; Newman-Keuls p<.001 for BTBR versus B6 at pnd 2, 4, 6, 8 and 10; e) bar holding: F(6,108) = 0.24, p<.001; Newman-Keuls p<.001 for BTBR versus B6 at pnd 6 and 8; f) cliff aversion: F(6,108) = 2.3, p<.05; Newman-Keuls p<.001 for BTBR versus B6 at pnd 4, 6, 8 and 12; Newman-Keuls p<.05 at pnd 10; g) forelimb placing: F(5,90) = 2.3, p<.05; Newman-Keuls p<.01 for BTBR versus B6 at pnd 4 and 6. BTBR pups showed a greater response to an acoustic stimulus, as shown by the auditory startle measurements on pnd 12 [strain×age interaction: F(3,54) = 0.51, p<.001; Newman-Keuls p<.001 for BTBR versus B6, Figure 8H], a time point at which pinnae detachment differed between strains.

#### Homing test

**Figure 9 pone-0003067-g009:**
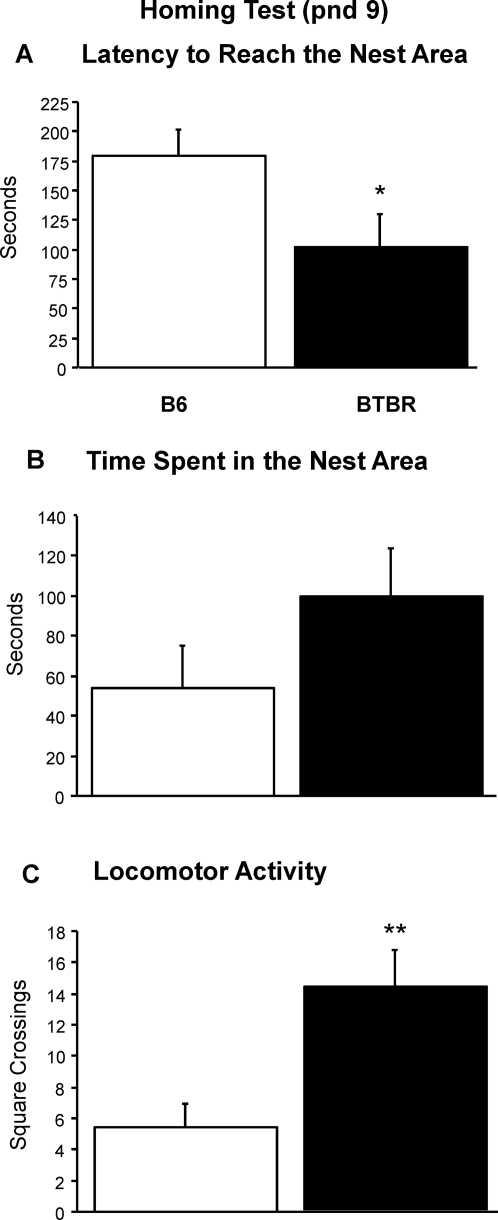
Pup homing test performed at pnd 9. Strains differed on A) latency to reach the area containing nest litter from their own home cages, B) Time spent in the nest area and C) general locomotor activity. *p<.05 for B6 vs. BTBR on latency; no significant difference for time spent in the nest area; **p<.01 for locomotor activity.

A significant strain effect was found on latency to reach the area containing the nest litter [Mann-Whitney, p = .03]. BTBR pups did not spend significantly more time in the nest area [F(1,18) = 1.9, p = 0.17]. A significant strain effect was found on general locomotor activity, indicating that the BTBR pups were more active than B6 pups [F(1,18) = 10. 4, p = .005] (Figure 9).

#### Open field test

**Figure 10:**
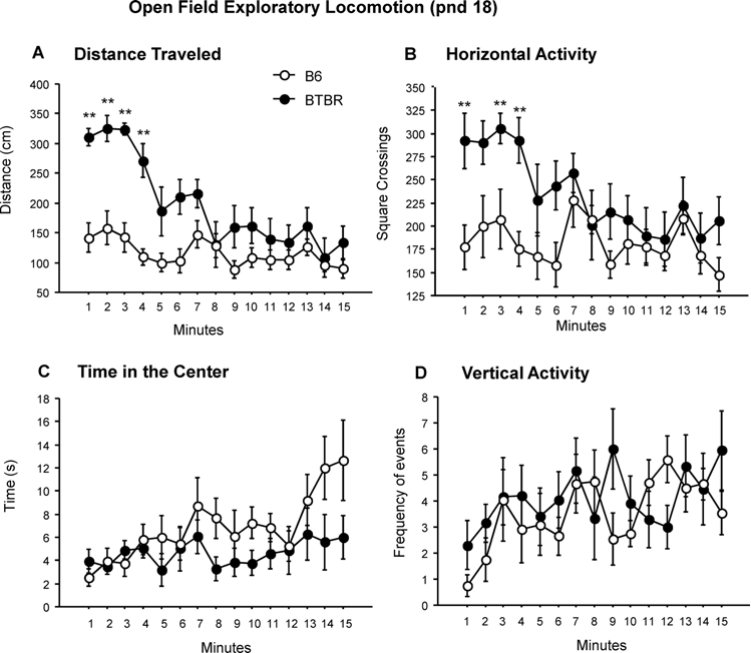
Open field activity was assayed at pnd 18. BTBR was initially more active higher than B6 on A) distance travelled and B) horizontal activity. There were no significant strain differences on C) center time and D) vertical activity in the open field arena. **p<.01 for B6 vs. BTBR.

Locomotor activity of 18-day-old pups in a non-social empty novel open field was initially higher in BTBR than in B6 [strain×minutes interaction: F(14,252) = 3.3, p<.0001; Newman-Keuls p<.001 for BTBR versus B6 in the first 4 minutes of the test] but subsequently, the two strains showed similar activity levels after habituation (Figure 10). The same profile was seen for horizontal activity where the BTBR mice showed a higher activity level [strain effect, F(1,18) = 8.7, p<.01; Newman-Keuls p<.001 for BTBR versus B6 in the first, third and fourth minute of the test].

Center time and vertical activity in the open field arena did not differ between strains [center time: strain×minutes interaction, F(14,252) = 1.2, p = .29; vertical activity: F(14,252) = 1.4, p = .17 ].

## Discussion

BTBR is a minimally characterized inbred strain of mice that displays deficits in social behaviors and high levels of repetitive self-grooming, traits relevant to the first and third diagnostic symptoms of autism [40]–[44]. We now report unusual properties of vocalizations in BTBR. BTBR pups separated from their mothers and siblings at postnatal days 2, 4, 6, 8, and 12 emitted significantly more calls, and those calls were of significantly longer duration than standard B6 mice. Total number of calls was higher in BTBR as compared to two other strains as well, 129X1, which is a close genetic relative, and FVB/NJ, which is close in body weight. In addition, BTBR calls showed smaller peak frequencies than the three other strains at pnd 6 and 8 and higher peak amplitudes than B6 at pnd 4, 6, 8 and 12, indicating overall qualitative differences as compared to the other strains. No differences were detected in body temperature after USV recording, excluding the possibility of unusual thermoregulation in BTBR.

We further investigated the specific types of calls emitted by each of the four inbred strains. Waveform patterns of the separation calls were classified into multiple categories of identified syllables emitted by BTBR, B6, 129X1 and FVB/NJ pups at postnatal age 8. Ten specific categories of calls were detected, designated as complex, harmonics, two-syllable, upward, downward, flat, chevron, short, composite and frequency steps, consistent with previous reports [21], [22], [24], [54]. A detailed analysis of the probability of vocalizations revealed that 8-day-old B6, FVB/NJ and 129X1 pups emitted a wide repertoire of calls, including high numbers of frequency steps and complex calls. BTBR pups emitted a narrower repertoire of calls, which included high levels of harmonics and composites, but minimal numbers of chevron-shaped syllables, upward, downward, and short calls.

Mouse pup calls incorporate some properties that suggest they could serve some of the same functions as the crying of human babies, especially their ability to elicit parental retrieval behaviors [11], [12], [55]–[61]. These studies indicated that most of the separation emissions carry the decisive acoustic and neurophysiological features for releasing maternal behavior, and thus are likely to be effective in communication [62]–[64]. Together with indications of left-hemisphere dominance of call perception in mice [65], these studies provide preliminary evidence for similar neuroanatomical and neurophysiological mechanisms mediating the perception of vocalizations in mice and humans.

Holy and Guo [23] first reported that vocalizations of adult male mice (C57BL/6×DBA/2J F1 cross) are more complex than previously known, and share characteristics of birdsong. Mouse vocalizations consist of several categories, i.e. different syllable types, whose temporal sequencing includes the utterance of repeated phrases. Consistent with the study of song production by adult mice, Panksepp and coworkers [24] found that many USVs in adolescent B6 and BALB/cJ mice were frequency-modulated, occurring in repetitive bouts separated by periods of silence. These adolescent vocalizations were remarkably complex, with a significant effect of genotype on each distinct USV category that was classified (upward, downward, chevron, complex and punctuated calls). Further, in adult mice, call categories and frequencies varied across stages of sexual behaviors, and were influenced by amphetamine treatments and gene disruptions [8].

Inspired by these recent reports, we classified every call emitted by four strains of mouse pups into ten categories, to search for strain differences. Our 8 day old B6 mice showed similar percentages of upward- and downward-modulated calls as compared to 30 day old B6 mice [24] but higher percentages of chevrons and complex calls. Differences in age, social setting, analysis parameters, and the use of five versus ten distinct categories may explain the discrepancies in percentages reported by Panksepp and coworkers [24] and the present data. Moreover, the mean duration and peak frequencies of these types of calls are not identical in 8 day and 30 day old B6 mice, most likely due to the different ages of the subjects and differences in the test conditions. Branchi and coworkers [21] previously reported a spectrographic characterization of eight day old outbred CD-1 mice, isolated from their mothers and littermates. CD-1 pups emitted a wide spectrum of USVs classified into five categories (flat, complex, frequency steps, short and composite). As compared to the present results with B6, 129X1, and FVB/NJ, the eight day old CD-1 emitted a higher percentage of frequency steps and complex calls but low numbers of flat, short and complex calls.

Thus, vocalization categories or syllables in B6, F1 BXD, BALB/cJ, and CD-1 seem to be generally similar to each other, to the extent that comparisons can be made of results from different laboratories. BTBR appears to be an outlier strain on both higher number of calls and restricted number of categories of calls. BTBR has been proposed as a mouse model of autism, because of its low levels of sociability, juvenile play, and social transmission of food preference, and its high levels of repetitive self-grooming, resistance to change in a spatial habit in the Morris water maze, and poor shift performance in a holeboard task [40]–[44], [66]. The present findings of unusual categories or syllables of vocalizations by BTBR may offer face validity to some forms of the second diagnostic symptom of autism, impaired communication. For example, some infants and young children later diagnosed with autism make atypical vocalizations. Instead of cooing and babbling, they may hum or grunt for extended periods, fail to add inflections into speech patterns, repeat “pop up” words out of context, squeal stereotypically, and laugh inappropriately [45], [67]–[70]. Others may be very irritable, cry for long periods of time, and be difficult to console [45]. More crying in these babies may be similar to the higher number of separation calls in BTBR pups, that were emitted at louder amplitudes than B6 beginning at pnd 4, and were primarily the harmonic syllable. In 81% of their 3633 calls, BTBR used only 4 of the 10 call types (harmonics, two-syllable, composite, and frequency steps). In contrast, each of the other three strains used a wider variety of the call types of their calls (2333 B6 calls, 1806 129X1 calls, 2575 FVB/NJ calls analyzed).

In rodent models of neurodevelopmental disorders, it is critical to conduct behavioral phenotyping during the early developmental period in order to document the precise onset of symptoms, identify transient signs, and provide a basis for the timing of early intervention [71], [72]. BTBR reached many developmental milestones earlier than B6, an inbred strain that is commonly used in behavioral genetics and for breeding targeted gene mutations. Faster growth and development in BTBR was seen in a fully developed righting reflex response by pnd 2 as compared to pnd 6 in B6, the full negative geotaxis response acquired by pnd 4 in BTBR versus pnd 8 in B6, and the more rapid growth of the body and tail length, earlier detachment of the pinnae, opening of the eyes, and incisor eruption in BTBR than B6. Faster acquisition of developmental milestones in BTBR may represent an unusual neurodevelopmental profile. Along with the larger head circumference at early ages in autism, accelerated growth of body length and higher weights were reported in three clinical studies [73]–[75]. However, it is important to note the possibility that B6 is the outlier on some measures of early neurodevelopment. The righting reflex developed most slowly in B6 as compared to the FVB/NJ and 129X1 strains which were more similar to BTBR. Body weights of B6 were similar to 129X1, while body weights of FVB/NJ were similar to BTBR.

The homing test incorporates more complex measures of interest in social odors, cognitive abilities for differentiating own versus other home cage odors, and the sensory and motor abilities required to navigate toward litter containing the pup's own home cage odor. In the homing test, 9-day-old BTBR pups showed shorter latencies to reach the area containing nesting material from their own home cage, suggesting more rapid development of social olfactory and cognitive abilities as compared to B6 pups of same age. However, data relative to number of squares crossed during the homing test, and distance traveled during the open field test, showed that BTBR had higher general activity than age-matched B6. It is possible that the shorter latencies on the homing test were related to the higher level of exploratory activity in BTBR.

From our results, it appears that qualitative as well as quantitative analyses will be useful in understanding the content of calls emitted during ultrasonic vocalization production in mice, to compare syllable categories from different strains or genotypes. It is interesting to speculate that variability in the types of syllable categories emitted by each strain represent “dialects” specific to each inbred strain. Similar experiments using many more inbred strains of mice will be needed, at different ages and in different environmental situations, to make definitive statements about strain variations in complex vocal repertoires. Our preliminary findings raise the notion that a large set of unusual syllable patterns in a mouse strain could be analogous to a distinct “dialect”, “vocabulary” or “language” in that strain. However, an interpretation of “language” implies a communication function for the vocalizations. Detailed investigations of calls emitted by one mouse, call responses emitted by another mouse, and the resulting changes in behavior, are needed to conclude that communication has occurred. Playback experiments may be useful to discover whether mice actually communicate meaningful information to each other using ultrasonic vocalizations. If so, an accurate analysis of ultrasonic emissions could provide a reliable assay to model the second diagnostic symptom of autism, impaired communication, for use in identifying genetic and environmental causes of autism, and evaluating proposed treatments.

## Materials and Methods

### Behavioral observations

BTBR, B6, FVB/NJ, and 129X1 breeding pairs were purchased from The Jackson Laboratory (Bar Harbor, ME). Mice were housed in standard wire-topped Plexiglas cages (42 cm×27 cm×14 cm) in a temperature and humidity controlled vivarium, with food and water available ad libitum, with lights on from 19:00 to 7:00. Behavioral testing was conducted between 9.30 and 14.00 h, during the dark phase of the circadian cycle. All procedures followed NIH guidelines for the care and use of laboratory animals, and were approved by the NIMH Animal Care and Use Committee. Ten days after pairing for breeding, the females were individually housed and subsequently inspected daily at 9:30 for pregnancy and delivery. The day of birth was considered as postnatal day (pnd) 0.

Pups were tattooed on the paw with animal tattoo ink (Ketchum permanent Tattoo Inks green paste, Ketchum Manufacturing Inc., Brockville ON Canada) by loading the ink into a 30G hypodermic needle and inserting the ink subcutaneously through the needle tip into the center of the paw. The procedure was performed at two days of age, immediately after behavioral testing. The procedure causes only minor brief pain and distress and does not require the use of anesthesia.

### Ultrasonic vocalizations in separated pups

Litters chosen for testing contained more than seven pups for BTBR (7.8±1.05), B6 (7.1±0.58) and FVB/NJ (7.5±0.34), and more than five pups for 129X1 (5.5±0.40; a strain known for small litters). One female and one male from each litter of BTBR, B6, FVB/NJ and 129X1 mice (n = 10 litters each strain) were used for baseline measurements of the ultrasonic vocalizations from pnd 2 to 12. Body weights and body temperatures of pups were measured after the ultrasonic vocalization test on pnd 2, 4, 6, 8 and 12. On each day of testing, each pup was placed into an empty plastic container (diameter, 5 cm; height 10 cm), located inside a sound-attenuating styrofoam box, and assessed for USVs during a five minute test. At the end of the five minute recording session, each pup was weighed and its axillary temperature measured by gentle insertion of the thermal probe in the skin pocket between upper foreleg and chest of the animal for about 30 seconds (Microprobe digital thermometer with mouse probe, Stoelting Co., Illinois, USA). No differences in patterns of calling were detected in a comparison of male and female pups, therefore data were collapsed across sex.

An Ultrasound Microphone (Avisoft UltraSoundGate condenser microphone capsule CM16, Avisoft Bioacoustics, Berlin, Germany) sensitive to frequencies of 10–180 kHz, recorded the pup vocalizations in the sound-attenuating chamber. The microphone was placed through a hole in the middle of the cover of the styrofoam sound-attenuating box, about 20 cm above the pup in its plastic container. The temperature of the room was maintained at 22±1°C. Vocalizations were recorded using Avisoft Recorder software (Version 3.2). Settings included sampling rate at 250 kHz; format 16 bit. For acoustical analysis, recordings were transferred to Avisoft SASLab Pro (Version 4.40) and a fast Fourier transformation (FFT) was conducted. Spectrograms were generated with an FFT-length of 1024 points and a time window overlap of 75% (100% Frame, Hamming window). The spectrogram was produced at a frequency resolution of 488 Hz and a time resolution of 1 ms. A lower cut-off frequency of 15 kHz was used to reduce background noise outside the relevant frequency band to 0 dB. Call detection was provided by an automatic threshold-based algorithm and a hold-time mechanism (hold time: 0.01 s). An experienced user checked the accuracy of call detection, and obtained a 100% concordance between automated and observational detection. Parameters analyzed for each test day included number of calls, duration of calls, qualitative and quantitative analyses of sound frequencies measured in terms of frequency and amplitude at the maximum of the spectrum.

Waveform patterns of calls were examined in depth in twenty sonograms collected from every strain, one from each of the pups tested. The sonograms were one minute in length and selected from recordings at postnatal day 8. We classified 3633 BTBR calls, 2333 B6 calls, 1806 129X1 calls and 2575 FVB/NJ calls. Each call was identified as one of 10 distinct categories, based on internal pitch changes, lengths and shapes, using previously published categorizations [21], [22], [24]. Classification of USVs included ten waveform patterns described below, and illustrated visually in Figure 2 and S1 and as audiofiles (Sounds S1, S2, S3, S4, S5, S6, S7, S8, S9, S10) in Supporting Information.

1. *Complex* calls displayed one syllable containing two or more directional changes in pitch, each ≥6.25 kHz.
2. *Harmonics* displayed one main call, resembling the complex calls, but with additional calls of different frequencies surrounding the main call.
3. *Two-syllable* calls consisted of two components: a main call (flat or downward) with an additional punctuated component towards the end.
4. *Upward*-modulated calls exhibited a continuous increase in pitch that was ≥12.5 kHz, with a terminal dominant frequency at least 6.25 kHz more than the pitch at the beginning of the vocalization.
5. *Downward*-modulated calls exhibited a continuous decrease in pitch that was ≥12.5 kHz, with a terminal dominant frequency at least 6.25 kHz less than the pitch at the beginning of the vocalization.
6. *Flat* calls displayed a constant beginning and the ending of the pitch frequency remained constant (≤3 kHz of each other).
7. *Chevron* calls resembled an ‘inverted-U’, which was identified by a continuous increase in pitch ≥12.5 kHz followed by a decrease that was ≥6.25 kHz.
8. *Short* calls were punctuated and shorter than 5 ms.
9. *Composite* calls were formed by two harmonically independent components, emitted simultaneously.
10. *Frequency steps* were instantaneous frequency changes appearing as a vertically discontinuous “step” on a spectrogram, but with no interruption in time.

Inter-rater reliability in scoring the call categories was 98%. Call category data were subjected to two different analyses: a) strain-dependent effects on the frequency and duration of the vocalizations emitted by each subject at pnd 8 b) strain-dependent effects on the probability of producing calls from each of the ten categories of USV, as described below under Statistical analysis.

### Developmental Milestones

To avoid potential confounds from using previously handled animals, a second cohort of B6 and BTBR pups was bred for the assays of developmental milestones, homing, and open field activity. One female and one male from each litter of BTBR and B6 mice (n = 10 litters each strain) were tested from pnd 2 to 18. Pups were transferred to a cage filled with clean bedding placed over a heating pad to maintain the temperature at 35°C. Every other day from pnd 2 to 14, pups were weighed to the nearest 0.01 g and their body and tail lengths were measured. Fur development, day of eyelid opening, pinnae detachment and incisor eruption were also recorded.

Pups were tested according to a slightly modified Fox battery [76]–[78]. The tests were conducted during the light phase of the circadian cycle, between 10:00 and 15:00 h. Each subject was tested at approximately the same time of day. Reflexes and responses were scored in the following order:

1. *Righting reflex*: pup turns over with all four feet on the ground when placed on its back.
2. *Negative geotaxis*: pup turns approximately 180° to either side when placed head down on a wire mesh screen (4×4 mm) held at a 45° angle.
3. *Cliff aversion*: pup withdraws from the edge of a flat surface when its snout and forepaws are placed over the edge of a table.
4. *Forelimb grasping reflex*: pup grasps the shaft of a toothpick when the forepaw is stroked.
5. *Forelimb placing reflex*: pup raises and places its forepaw on the surface of the edge of an object when stroked on the *dorsum* of the paw.
6. *Vibrissa placing reflex*: pup places its forepaw onto a cotton swab stroked across its vibrissae.
7. *Auditory startle*: pup reacts to acoustic stimuli (snapping of the fingers) by a startle response.
8. *Level screen tests*: pup holds onto a wire mesh screen when it is dragged across it horizontally by the tail.
9. *Screen climbing test*: pup climbs up the vertical wire mesh screen (90° angle) using both fore- and hind-paws. Maximal response is scored when the subject reaches the top of the vertical screen (10×10 cm), which usually takes about 5 s.
10. *Bar holding*: pup grasps a small wire bar by its forelimbs. Bar holding was scored as present if the pup is able to hang suspended for 10 s.

Latencies were measured in seconds, using a stopwatch for righting reflex, negative geotaxis and bar holding. Other somatic and behavioral variables were rating semi-quantitatively: 0 = no response/reaction, 1 = slight response/reaction, 2 = incomplete response/reaction, and 3 = a complete adult-like response. Investigators were trained until the inter-observer reliability was greater than 95%. Unless otherwise noted, absence of a milestone was scored as zero if the mouse did not exhibit the behavior within 60 s.

#### Homing test (pnd 9)

On pnd 9, the litter was separated from the dam and kept for 30 min in one holding cage. The cage was placed on a heating pad set at a temperature of 35°C to maintain normal body temperature of the pups in the nest. Individual pups were then transferred to a Plexiglas cage (36 cm×22.5 cm, walls 10 cm high). Wood shavings from the home cage were evenly spread on one side (14 cm×22.5 cm, nest area) while the rest of the cage was covered with clean bedding. The pup was placed in the middle of the Plexiglas cage and videorecorded for four minutes. The floor of the arena was virtually subdivided into squares of 7 cm×7 cm each, to enhance scoring of locomotor activity from the video digital DVDs, using Noldus Observer 5.0 software (Noldus Information Technology, Leesburg, VA). Homing performance was scored for latency to reach the area containing nest litter, time spent in the area containing nesting litter and locomotor activity by square crossings.

#### Open field test (pnd 18)

One male and one female from each litter, which were not used in the developmental milestones and homing assessments, were tested for locomotor activity on pnd 18. General exploratory locomotion in a novel environment was tested by placing pups in a VersaMax Animal Activity Monitoring System (AccuScan Instruments, Columbus, OH, USA) for a 15-min test session. To compensate for the relatively small size of 18-day-old-male mice, the VersaMax vertical sensor was adjusted to the lowest setting of 7 cm, and the floor of the open field arena was elevated by 1.0 cm, so that the final height of the vertical sensor was 6.0 cm above the floor of the arena. The testing room was illuminated with a single 25-W red lamp and kept at a similar temperature as the colony room. The test was carried out between 10.00 and 13.00 h.

#### Statistical analysis

In the neonatal studies, statistical analysis based on litters as statistical units and pups as repeated trials within each litter was performed. Since no sex differences were detected, data were collapsed across sex.

A mixed-model Analysis of Variance (ANOVA) with Repeated Measures was used to analyze a) neonatal USVs and developmental milestone responses, with the strain as factor and postnatal day as the repeated measures, b) homing responses, frequency and duration, c) open field activity, with session minutes as the repeated measure. Nonparametric analysis (Mann Whitney test) was used to analyze latencies in the developmental milestones and homing tests. Post-hoc comparisons were performed using Newman Keuls test only when a significant *F*-value was determined. For all comparisons, significance was set at *P*<0.05.

An ANOVA with repeated measures was performed to analyze strain-dependent effects on the vocalizations data, with the strain as factor and call categories as the repeated measures. Probability of vocalizations within strain was calculated as number of calls in each category for each subject/total number of calls analyzed in each subject and standardized by angular transformation. To better analyze strain differences within call categories, an ANOVA was performed for each category and Newman-Keuls comparison was followed by Bonferroni correction.

## Supporting Information

Figure S1 Complex vocal repertoire of mouse pup separation calls. Audioclips representing examples of the ten distinct categories of calls are provided (Sounds S1, S2, S3, S4, S5, S6, S7, S8, S9 and S10). Recordings were collected and converted from mouse's ultrasonic range to the human hearing range by Avisoft software. In order to appreciate the waveforms in greater detail, a selected call of each subtype was converted from the sample rate of 250 kHz in the original wav file to 11.025 kHz, resulting in a slower speed for human listening.
(0.20 MB PPT)

Sound S1 Chevron vocalization from a male B6 pup at pnd 8, corresponding to the sonogram in Figure S1 labeled “chevron”.
(0.00 MB MP3)

Sound S2 Harmonic vocalization from a male BTBR pup at pnd 8, corresponding to the sonogram in Figure S1 labeled “harmonic”.
(0.02 MB MP3)

Sound S3 Upward vocalization from a male B6 pup at pnd 8, corresponding to the sonogram in Figure S1 labeled “upward”.
(0.01 MB MP3)

Sound S4 Downward vocalization from a male B6 pup at pnd 8, corresponding to the sonogram in Figure S1 labeled “downward”.
(0.01 MB MP3)

Sound S5 Complex vocalization from a male B6 pup at pnd 8, corresponding to the sonogram in Figure S1 labeled “complex”.
(0.01 MB MP3)

Sound S6 Short vocalization from a male B6 pup at pnd 8, corresponding to the sonogram in Figure S1 labeled “short”.
(0.00 MB MP3)

Sound S7 Two-Syllable vocalization from a male B6 pup at pnd 8, corresponding to the sonogram in Figure S1 labeled “two-syllable”.
(0.01 MB MP3)

Sound S8 Flat vocalization from a male B6 pup at pnd 8, corresponding to the sonogram in Figure S1 labeled “flat”.
(0.01 MB MP3)

Sound S9 Composite vocalization from a male B6 pup at pnd 8, corresponding to the sonogram in Figure S1 labeled “composite”.
(0.01 MB MP3)

Sound S10 Frequency step vocalization from a male B6 pup at pnd 8, corresponding to the sonogram in Figure S1 labeled “frequency step”.
(0.02 MB MP3)

Table S1 Body Temperature measured after pup separation vocalizations recording.
(0.02 MB XLS)
