# Supplementary material for: Unusual Repertoire of Vocalizations in the BTBR T+tf/J Mouse Model of Autism
Source: PLoS One. 2008 Aug 27;3(8):e3067. doi: 10.1371/journal.pone.0003067 (PMC2516927; doi:10.1371/journal.pone.0003067)
Supplement: Figure S1 — Complex vocal repertoire of mouse pup separation calls. Audioclips representing examples of the ten distinct categories of calls are provided (Sounds S1, S2, S3, S4, S5, S6, S7, S8, S9 and S10). Recordings were collected and converted from mouse's ultrasonic range to the human hearing range by Avisoft software. In order to appreciate the waveforms in greater detail, a selected call of each subtype was converted from the sample rate of 250 kHz in the original wav file to 11.025 kHz, resulting in a slower speed for human listening. (0.20 MB PPT) [file pone.0003067.s001.ppt]

## Slide 1
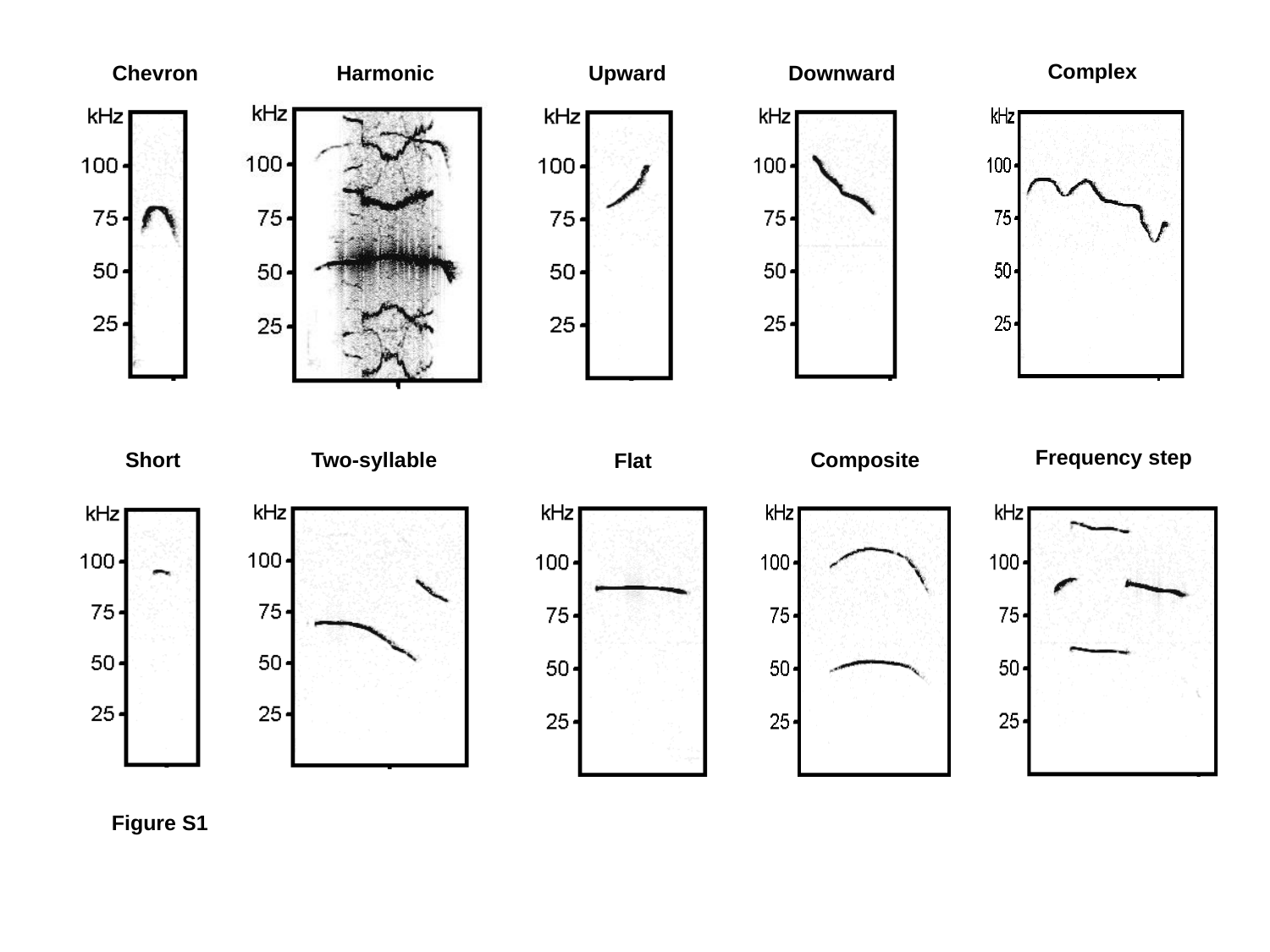

Complex
Chevron
Harmonic
Upward
Downward
Frequency step
Short
Two-syllable
Composite
Flat
Figure S1
